# Machine-learning based prediction and analysis of prognostic risk factors in patients with candidemia and bacteraemia: a 5-year analysis

**DOI:** 10.7717/peerj.13594

**Published:** 2022-06-15

**Authors:** Yali Gao, Mingsui Tang, Yaling Li, Xueli Niu, Jingyi Li, Chang Fu, Zihan Wang, Jiayi Liu, Bing Song, Hongduo Chen, Xinghua Gao, Xiuhao Guan

**Affiliations:** 1 Department of Dermatology, The First Affiliated Hospital, Sun Yat-sen University, Guangzhou, Guangdong, China; 2 Department of Dermatology, The First Hospital of China Medical University, Shenyang, Liaoning, China; 3 School of Dentistry, Cardiff University, Cardiff, United Kingdom

**Keywords:** Candidemia, Bacteriaemia, Epidemiology, Prognosis, Machine learning

## Abstract

Bacteraemia has attracted great attention owing to its serious outcomes, including deterioration of the primary disease, infection, severe sepsis, overwhelming septic shock or even death. Candidemia, secondary to bacteraemia, is frequently seen in hospitalised patients, especially in those with weak immune systems, and may lead to lethal outcomes and a poor prognosis. Moreover, higher morbidity and mortality associated with candidemia. Owing to the complexity of patient conditions, the occurrence of candidemia is increasing. Candidemia-related studies are relatively challenging. Because candidemia is associated with increasing mortality related to invasive infection of organs, its pathogenesis warrants further investigation. We collected the relevant clinical data of 367 patients with concomitant candidemia and bacteraemia in the first hospital of China Medical University from January 2013 to January 2018. We analysed the available information and attempted to obtain the undisclosed information. Subsequently, we used machine learning to screen for regulators such as prognostic factors related to death. Of the 367 patients, 231 (62.9%) were men, and the median age of all patients was 61 years old (range, 52–71 years), with 133 (36.2%) patients aged >65 years. In addition, 249 patients had hypoproteinaemia, and 169 patients were admitted to the intensive care unit (ICU) during hospitalisation. The most common fungi and bacteria associated with tumour development and Candida infection were *Candida parapsilosis* and *Acinetobacter baumannii*, respectively. We used machine learning to screen for death-related prognostic factors in patients with candidemia and bacteraemia mainly based on integrated information. The results showed that serum creatinine level, endotoxic shock, length of stay in ICU, age, leukocyte count, total parenteral nutrition, total bilirubin level, length of stay in the hospital, PCT level and lymphocyte count were identified as the main prognostic factors. These findings will greatly help clinicians treat patients with candidemia and bacteraemia.

## Introduction

Candidemia and bacteraemia frequently occur in hospitalised or critical care patients with unfavourable prognosis and high mortality (Raoult & Richet, 2011; Bloos et al., 2013; Heimann et al., 2015; Kullberg & Arendrup, 2015; Logan, Martin-Loeches & Bicanic, 2020). Candidemia is diagnosed in >250,000 people annually worldwide and causes >50,000 deaths (Arendrup, 2010). According to a population-based study from the United States of America (USA) and a database-based systematic analysis from Europe, bacteraemia is the fourth leading cause of mortality (following cardiac diseases, combined lung and larynx cancers and cerebrovascular diseases) (Jensen et al., 2011; Sogaard et al., 2011). The mortality rate of candidemia was 29% according to a population-based study from the USA, 31% according to a study from Spain, 54% according to a multi-centre study from Brazil and 60% according to a survey conducted in South Africa (Colombo et al., 2006; Cleveland et al., 2012; Kreusch & Karstaedt, 2013; Puig-Asensio et al., 2014). In addition, on the basis of a study from the University of Pennsylvania, catheter-associated bacteraemia was discovered to be the 12th leading cause of death in USA (Umscheid et al., 2011). Furthermore, the distribution and prevalence of different *Candida* species differ according to regional discrepancies and patient populations (Pappas et al., 2018). Among fungal infections, *Candida albicans* infection is the most prevalent infection leading to death; however, the incidence of non-*albicans* candidemia has increased over the past decades worldwide (Pfaller et al., 2011; Castanheira et al., 2016; Lamoth et al., 2018). Moreover, the number of cases of *Candida albicans* infection dramatically decreased during the past decade in the USA and is less than half of that reported previously (Lockhart et al., 2012; Matsumoto et al., 2014; Pfaller, Jones & Castanheira, 2014). Because candidemia is frequently reported and *Candida* is the third most common causative agent of infection in intensive care units (ICUs) worldwide (17%), medical support and intensive treatment are challenging (Vincent et al., 2009; Colombo et al., 2014; Lortholary et al., 2014; Chakrabarti et al., 2015). Bloodstream infections often lead to severe diseases with high morbidity and mortality and can be acute or chronic (Opota et al., 2015). Candidemia and bacteraemia are associated with a heavy socioeconomic burden and widespread prevalence (Martinez & Wolk, 2016). Studies reporting on the epidemiological features and risk factors of candidemia and bacteraemia are limited; therefore, further investigation and integration are required.

Machine learning is a rapidly developing technique that is widely used for analysing medical information and making clinical decisions. Programming algorithms through machine learning can define rules based on extensive data and interpret unknown relationships between factors (Jordan & Mitchell, 2015; Peiffer-Smadja et al., 2020). Random forest (RF), logistic regression (LR) and support vector machine (SVM) are widely used classification tools in bioinformatics and medical fields. RF is a supervised tree-based ensemble machine learning methodology, whereas SVM is a nonparametric, supervised and kernel-based statistical learning approach (Saberioon et al., 2018). The relationship between one or more independent factors and a binary dependent variable can be estimated using logistic regression (Schober & Vetter, 2021). Epidemiological characteristics and risk factors can be processed, analysed and predicted by using machine learning. Therefore, we used machine learning methods in this retrospective study to analyse the clinical information and find out risk factors for patients with candidemia and bacteraemia.

## Methods

### Human ethics

This study was conducted in accordance with the declaration of Helsinki. It was approved by The Human Ethics Review Committee of the First Hospital of China Medical University (number: 2021-260). The ethics review board of the First Hospital of China Medical University exempted the acquisition of informed consent because it was a retrospective study. During data collection and preparation of the manuscript, all patients’ information was considered to be confidential.

### Patient selection

Patients were selected and data were collected as described in our previous study (Li et al., 2020). Specially, all data on *Candida* recovered from the blood of patients with invasive candidal infection were acquired (2008 version of EORTC/MSG criteria). We used the date when the first positive result of blood culture was obtained as the onset of candidemia and bacteraemia. The data set captured relevant information from the selected patients, including patient clinical features, risk factors for candidemia and bacteraemia, treatment and survival status at discharge, haematological diagnoses, *Candida* and bacterial test results, antifungal therapy and so on. The hospitalisation of each patient was regarded as an event. It should be noted that it was considered to be a new event once the patient was re-hospitalised and received new treatments.

### Definition

Persistent candidemia was defined as a condition in which the blood culture tests yielded positive results with the same *Candida* species after 7 days of initiating appropriate therapy.

### Microbiological test

The collected blood samples were cultured for 5 days, then we selected the positive blood samples and transferred them to blood AGAR plates. Fungal isolates and bacterial isolates were cultured at 35 °C for 48-72 h subsequently. We carried out gram staining and microscopic examination simultaneously. Strain identification was performed on a VITEK two Compact (Bio-Merieux SA, Marcy l’etoile, France), including fungal isolates and bacterial isolates. Drug susceptibility tests were performed followed the reagent instructions and the “national clinical test operating procedures” using the ATB FUNGUS three (Bio-Merieux SA, Marcy l’etoile, France). The ATB Fungus 3 yeast-like fungi was applied into a drug susceptibility test box, and the minimum inhibitory concentration (MIC) value was determined according to the CLSI m27-a3 and m27-s4 antifungal susceptibility test standards. *Candida* ATCC6258 and *Candida* albicans ATCC90028 were used as the quality control strains.

### Machine learning

We pre-processed the data, removed missing cases (missing data with 50% features) and filled the mean value of missing data. We performed five-fold cross-validation to analyze these data. The predictive power of the model was measured using the average area under the curve (AUC) of the receiver operating characteristic curve based on five-fold cross-validation process. In addition, random forest, logistic regression and support vector machine were used to develop the final prediction model. We applied the model of random forest to identify important features.

### Statistical analysis

Statistical analysis was performed using the SPSS 20.0 software. Non-normally distributed quantitative data were expressed as median and quartile ranges [M(P25, P75)] and the intergroup comparisons were performed using the Mann–Whitney test. Qualitative data were described by relative numbers and the intergroup comparisons were made using chi-square test.

## Results

### Clinical features of patients

A total of 367 patients with candidemia and bacteraemia were included in this study, 231 (62.9%) were male and 136 (37.1%) were female. The median age of all patients with candidemia was 61 years (range, 52–71 years), with 133 (36.2%) patients aged >65 years. Among all patients, 169 (46.0%) stayed in ICU during hospitalisation, 358 (97.5%) stayed in the hospital for >10 days and 224 (61.0%) had multiple hospitalisations within the past 2 years. Diseases found in patients were hypoproteinaemia (249 patients), solid tumours (182 patients), haematological malignancies (10 patients), diabetes (50 patients), renal failure (37 patients) and pancreatitis (37 patients). Other common relevant conditions contributing to candidemia included the long-term use of broad-spectrum antibiotics (196/367, 53.4%), recent surgery (<2 weeks ago) (198/367, 54.0%), invasive mechanical ventilation (143/367, 39.0%), urinary catheter insertion (285/367, 77.7%), gastric tube insertion (213/367, 58.0%), central venous catheter insertion (229/367, 62.4%), drainage catheter insertion (245/367, 66.8%) and total parenteral nutrition (298/367, 81.2%). In addition, 146 patients had persistent invasive mycosis. Detailed information is provided in Table S1.

**Figure 1:**
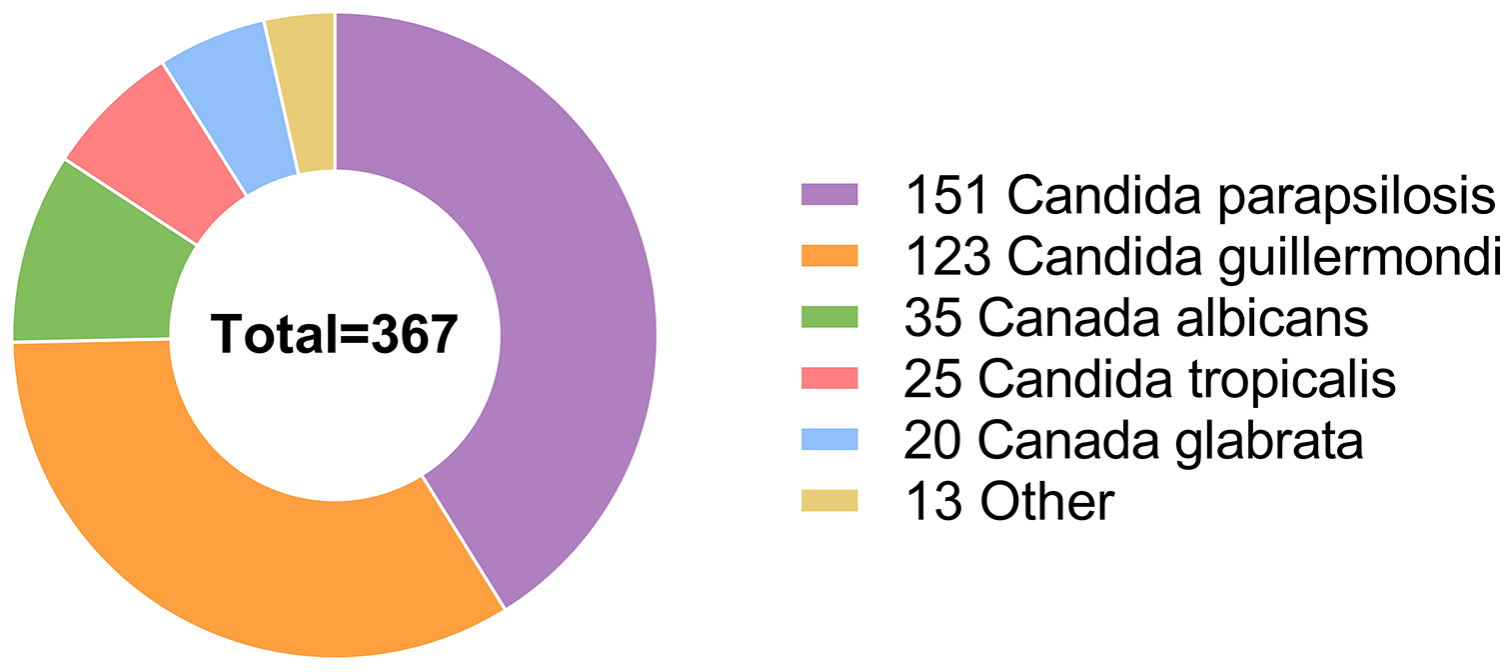
Among the 367 hospitalized patients, the most frequent infection agent was *Candida parapsilosis*, followed by Candida guilliermondii, *Candida albicans*, *Candida tropicalis*, *Candida glabrata*, and some other Candida species.

*Candida parapsilosis* was the most common causative agent affecting 151 patients. *Candida guilliermondi* was the causative agent in 123 patients, whereas other *Candida* species caused infections in a relatively small proportion of patients, including *Candida albicans* (35/367 patients), *Candida tropicalis* (25/367 patients), *Candida glabrata* (20/367 patients), *Cryptococcus neoformans* (9/367 patients), *Cadida lusitaniae* (2/367 patients), *Cadida krusei* (1/367 patients) and *Streptomyces* (1/367 patients) (Fig. 1).

**Figure 2:**
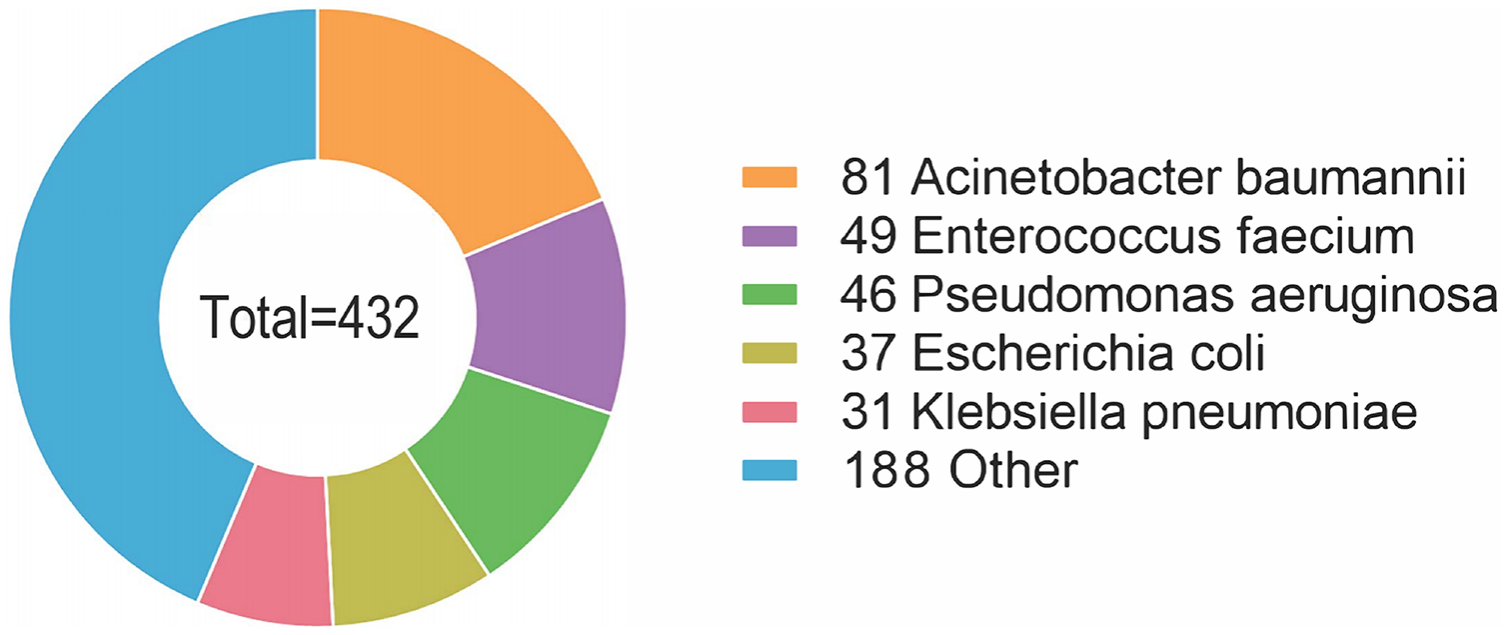
The most common species of bacteria was *Acinetobacter baumannii*, followed by *Enterococcus faecium*, *Pseudomonas aeruginosa*, *Escherichia coli*, *Klebsiella pneumoniae*, and some other species of bacteria.

Furthermore, 38 species of bacteria were isolated, with 16 (16/38, 42.1%) Gram-positive species and 22 (22/38, 57.9%) Gram-negative species. A total of 176 (176/367, 48.0%) patients were infected with Gram-positive bacteria, and 256 (256/367, 69.8%) patients were infected with Gram-negative bacteria. The most common species of bacteria was *Acinetobacter baumannii*, affecting 81 patients (81/367, 22.1%). *Enterococcus faecium* and *Pseudomonas aeruginosa* were also common species, affecting 49 (13.4%) and 46 (12.5%) patients, respectively, followed by *Escherichia coli* (37/367, 10.1%), *Klebsiella pneumoniae* (31/367, 8.4%), *Staphylococcus aureus* (29/367, 7.9%), *Staphylococcus epidermidis* (28/367, 7.6%), *Staphylococcus hominis* (21/367, 5.7%), *Stenotrophomonas maltophilia* (14/367, 3.8%), *Staphylococcus haemolyticus* (12/367, 3.3%), *Burkholderia cepacia* (10/367, 2.7%) and some other species of bacteria (Fig. 2). Detailed information is provided in Table S2.

### *In vitro* antifungal susceptibility test

**Figure 3:**
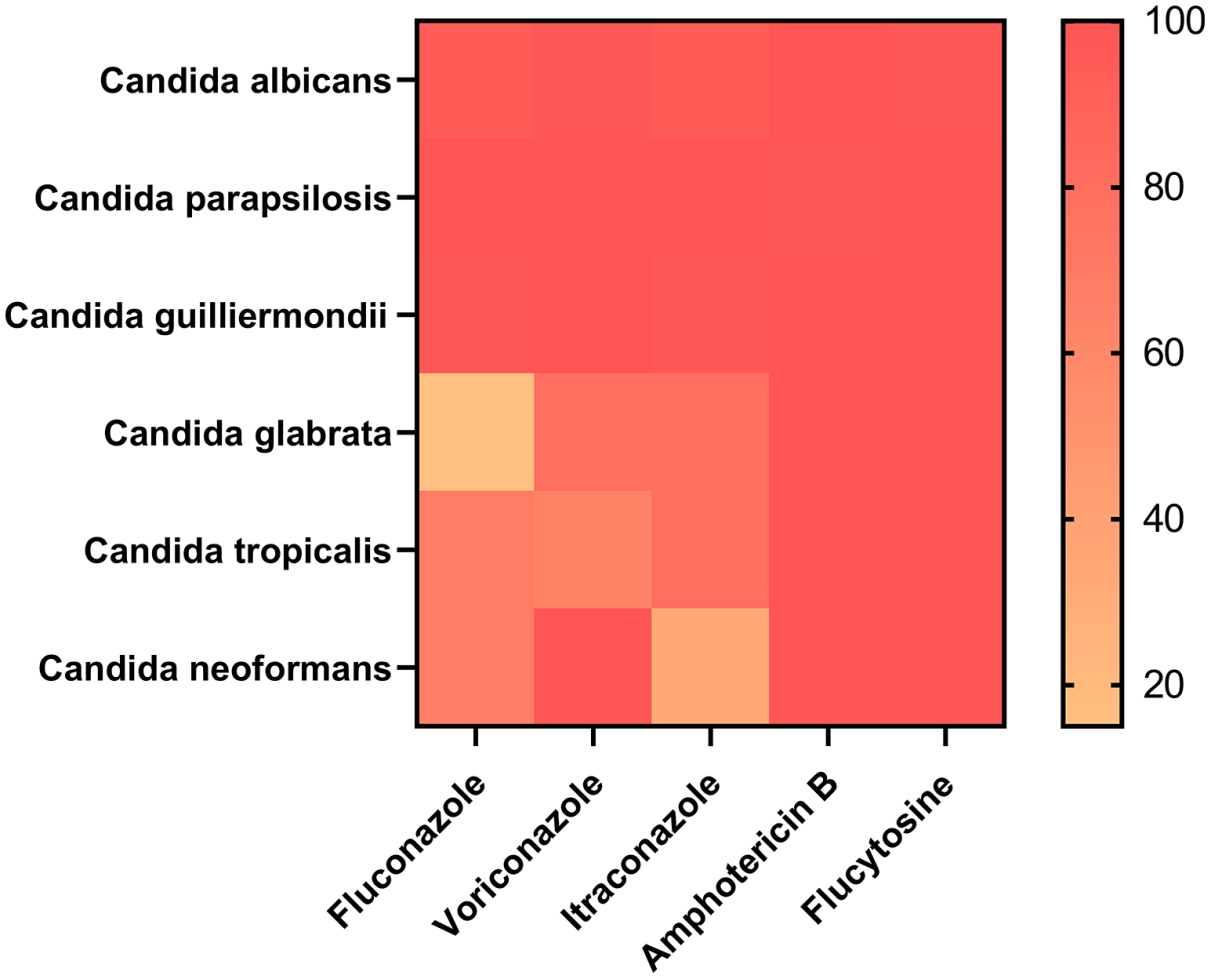
Amphotericin B and flucytosine are more effective drugs for the treatment of *Candida* infection.

Of the 367 patients, 353 patients with information on drug sensitivity were selected. Approximately 20 patients were resistant to at least one drug. Amphotericin B exhibited strong efficiency because all 353 patients were sensitive to it. A total of 352 patients were sensitive to flucytosine, with only one patient being resistant to it, which showed that amphotericin B and flucytosine may be potential drugs for treating *Candida* infection. In addition, 339 and 335 patients were sensitive to voriconazole and itraconazole, respectively. Moreover, 17 (17/20, 85%) patients with *Candida glabrata* infection showed strong dose-dependent drug sensitivity to fluconazole, which was not observed in any other groups, suggesting that fluconazole is a candidate drug for the treatment of *Candida glabrata* infection. However, 6 (6/25, 24%) patients with *Candida tropicalis* infection were resistant to fluconazole and voriconazole. Detailed information is provided in Fig. 3 and Table S3.

### Risk factors for *Candida albicans* and non- *Candida albicans* infections

Clinical information and statistical data are presented in Table 1. Approximately 52.11% of patients with non-*Candida albicans* infection and 31.42% of patients with *Candida albicans* infection had solid tumours. Furthermore, it was observed that a relatively large proportion (84.64%) of patients with non-*Candida albicans* infection was administered total parenteral nutrition; however, only 48.57% of patients with *Candida albicans* infection were administered total parenteral nutrition. In addition, 31.13% of patients with *Candida albicans* infection and 56.33% of patients with non-*Candida albicans* infection underwent a recent surgery (within 2 weeks). Urinary, central venous and drainage catheters were used in 79.22%, 64.16% and 71.39% of patients with non-*Candida albicans* infection, respectively; and 62.86%, 45.71% and 22.86% of patients with *Candida albicans* infection, respectively. As a result, patients with the catheters usage were more susceptible to non-*Candida albicans* infection. Furthermore, higher percentage of patients with *Candida albicans* infection (22.86%) were predisposed to endotoxic shock than that of patients with non-*Candida albicans* infection (10.54%).

**Table 1:** Risk factors for *Candida albicans* and non-*Candida albicans* infections.

	*Candida albicans* (%) (*n* = 35)	non-*Candida albicans* (%) (*n* = 332)	Statistic	*P* value
Male	20 (57.14%)	211 (63.55%)	0.747	0.455
Age (years)^a^	62.00 (45.50, 72.50)	61.00 (52.00, 71.00)	−0.152	0.879
Length of stay (days)^a^	33.00 (21.50, 67.00)	32.00 (23.00, 51.00)	0.101	0.920
Length of stay in ICU^a^	3.00 (0.00, 20.50)	0.00 (0.00, 11.25)	1.452	0.147
Solid tumor	11 (31.42%)	173 (52.11%)	2.327	0.020
Diabetes	4 (11.43%)	46 (13.86%)	0.398	0.691
Pancreatitis^b^	3 (8.57%)	34 (10.24%)	–	1
Total parenteral nutrition	17 (48.57%)	281 (84.64%)	5.194	<0.001
Renal failure	6 (17.14%)	31 (9.34%)	–	0.145
Recent surgery (within 2 weeks)	11 (31.13%)	187 (56.33%)	2.811	0.005
Use immunosuppressants^b^	3 (8.57%)	24 (7.23%)	–	0.733
ICU	20 (57.14%)	149 (44.88%)	1.384	0.166
Hypoproteinemia	25 (71.43%)	224 (67.47%)	0.477	0.633
Invasive mechanical ventilation	17 (48.57%)	126 (37.95%)	1.225	0.221
Urinary catheter	22 (62.86%)	263 (79.22%)	2.210	0.027
Gastric tube	17 (48.57%)	196 (59.04%)	1.193	0.233
Central venous catheter	16 (45.71%)	213 (64.16%)	2.142	0.032
Drainage catheter	8 (22.86%)	237 (71.39%)	5.797	<0.001
Endotoxic shock^b^	8 (22.86%)	35 (10.54%)	–	0.048
Multiple hospitalizations within 2 years (>2 times)	22 (62.86%)	202 (60.84%)	0.232	0.816
Persistent fungal infection	14 (40.00%)	132 (39.76%)	0.028	0.978
Serum albumin level^a^ (g/l)	26.20 (21.50, 31.25)	27.70 (23.90, 31.05)	−1.182	0.237
Serum creatinine level^a^ (μmol/L)	67.00 (49.50, 92.25)	59.00 (45.00, 79.00)	1.725	0.085
Leukocyte count^a^ (10^9^/l)	8.84 (6.94, 12.64)	6.46 (4.39, 9.80)	3.171	0.002
Total bilirubin level^a^ (μmol/l)	14.30 (8.95, 21.40)	13.25 (7.65, 24.00)	0.476	0.634
Neutrophil count^a^ (10^9^/l)	6.75 (4.63, 9.42)	5.13 (3.47, 8.06)	2.030	0.042
Lymphocyte count^a^ (10^9^/l)	0.75 (0.56, 1.14)	0.69 (0.45, 1.00)	1.375	0.169
CRP^a^ (mg/l)	106.00 (71.53, 166.50)	86.30 (47.35, 129.50)	1.520	0.128
PCT^a^ (ng/ml)	1.25 (0.52, 3.05)	0.50 (0.24, 1.39)	2.706	0.007

**Note:**

a Described by median and quartile, and the statistic was the Z value; other items were described as numbers (n - %) and the statistic was the χ2 value.

b Fisher χ2 value.

In patients with concomitant candidemia and bacteraemia, procalcitonin (PCT) levels, leukocyte and neutrophil counts were elevated, especially in patients with *Candida albicans* infection. Detailed information is provided in Table 1.

### Analysis of risk factors in patients with persistent and non-persistent *Candida* infections

The clinical information and statistical data of patients with persistent and non-persistent *Candida* infection are provided in Table 2. Persistent *Candida* infection was associated with prolonged hospital and ICU stays, total parenteral nutrition, recent surgery (within the past 2 weeks) and central venous catheter insertion. In addition, leukocyte, neutrophil and lymphocyte counts were significantly elevated in patients with persistent *Candida* infection, with statistically significant differences. Detailed information is provided in Table 2.

**Table 2:** Risk factors in patients with persistent and non-persistent candidal infections.

	Persistent candidal infection (%) (*n* = 146)	non-Persistent candidal infection (%) (*n* = 114)	Statistic	*P* value
Male	92 (63.01%)	73 (64.04%)	0.170	0.865
Age (years)^a^	62.00 (52.00, 72.75)	60.00 (51.00, 68.50)	1.297	0.195
Length of stay (days)^a^	40.50 (27.25, 66.75)	32.00 (24.00, 48.50)	3.241	0.001
Length of stay in ICU^a^	7.00 (0.00, 30.00)	0.00 (0.00, 8.00)	3.777	<0.001
Solid tumor	62 (42.47%)	59 (51.75%)	1.490	0.136
Diabetes^b^	24 (16.44%)	14 (12.28)	–	0.381
Pancreatitis^b^	16 (10.96%)	12 (10.53%)	–	1
Total parenteral nutrition	113 (77.40%)	100 (87.71%)	2.146	0.032
Renal failure^b^	22 (15.07%)	8 (7.02%)	–	0.051
Recent surgery (within 2 weeks)	65 (44.52%)	67 (58.77%)	2.281	0.023
Use immunosuppressants within the past 30 days^b^	11 (7.53%)	10 (8.77%)	–	0.820
Stay in ICU during hospitalization	86 (58.90%)	48 (42.11%)	2.689	0.007
Hypoproteinemia	108 (73.97%)	77 (67.54%)	1.135	0.256
Invasive mechanical ventilation	74 (50.68%)	46 (40.35%)	1.659	0.097
Urinary catheter	115 (78.77%)	86 (75.44%)	0.636	0.525
Gastric tube	89 (60.96%)	66 (57.89%)	0.500	0.617
Central venous catheter	107 (73.29%)	66 (57.89%)	2.610	0.009
Drainage catheter	98 (67.12%)	80 (70.18%)	0.526	0.599
Endotoxic shock^b^	24 (16.44%)	12 (10.53%)	–	0.207
Multiple hospitalizations within 2 years (>2 times)	99 (67.81%)	70 (61.40%)	1.074	0.283
Serum albumin level^a^ (g/l)	27.05 (23.10, 30.08)	27.85 (24.30, 32.25)	−1.453	0.146
Serum creatinine level^a^ (μmol/L)	59.00 (42.00, 85.50)	59.00 (47.00, 77.00)	−0.148	0.882
Leukocyte count^a^ (10^9^/l)	7.50 (5.37, 10.87)	5.86 (3.96, 8.80)	3.507	<0.001
Total bilirubin level^a^	13.75 (9.25, 29.20)	14.00 (7.40, 22.55)	0.808	0.419
Neutrophil count^a^ (10^9^/l)	6.28 (4.22, 8.67)	4.44 (2.89, 7.30)	3.515	<0.001
Lymphocyte count^a^ (10^9^/l)	0.77 (0.57, 1.10)	0.58 (0.42, 0.83)	3.378	<0.001
CRP^a^ (mg/ml)	92.00 (48.83, 130.50)	87.90 (58.18, 130.00)	−0.165	0.869
PCT^a^ (ng/ml)	0.54 (0.26, 1.19)	0.48 (0.26, 1.48)	0.164	0.870

**Note:**

a Described by median and quartile, and the statistic was the Z value; other items were described as numbers (n - %) and the statistic was the χ2 value.

b Fisher χ2 value.

### Analysis of risk factors in patients with single and multiple fungal infections

Of the 367 patients with candidemia and bacteraemia, 59 (16.1%) patients had multiple fungal infections, whereas 308 (83.9%) patients had a single fungal infection. Clinical information and statistical data are presented in Table 3.

**Table 3:** Analysis of risk factors in patients with single fungal infection and multiple fungal infections.

	single fungal infection (*n* = 308)	multiple fungal infection (*n* = 59)	Statistic	*P* value
Male	200 (64.94%)	31 (52.54%)	1.806	0.071
Age (years)^a^	61.00 (51.00, 69.00)	65.00 (53.00, 75.00)	−1.980	0.048
Length of stay (days)^a^	30.00 (22.00, 47.00)	55.00 (33.50, 95.00)	−5.193	<0.001
Length of stay in ICU^a^	0.00 (0.00, 8.00)	19.00 (0.00, 56.00)	−6.045	<0.001
Solid tumor	165 (53.57%)	16 (27.12%)	3.723	<0.001
Diabetes	32 (10.39%)	18 (30.51%)	4.127	<0.001
Pancreatitis^b^	33 (10.71%)	4 (6.78%)	–	0.481
Total parenteral nutrition	249 (80.84%)	49 (83.05%)	0.397	0.691
Renal failure	29 (9.42%)	8 (13.56%)	–	0.346
Recent surgery (within 2 weeks)	183 (59.42%)	15 (25.42%)	4.799	<0.001
Use immunosuppressants within the past 30 days^b^	26 (8.44%)	1 (1.69%)	–	0.098
Stay in ICU during hospitalization	125 (40.58%)	42 (71.19%)	4.324	<0.001
Hypoproteinemia	209 (67.86%)	40 (67.80%)	0.009	0.993
Invasive mechanical ventilation	105 (34.09%)	38 (64.41%)	4.374	<0.001
Urinary catheter	236 (76.62%)	49 (83.05%)	1.086	0.278
Gastric tube	174 (56.49%)	39 (66.10%)	1.370	0.171
Central venous catheter	182 (59.09%)	47 (79.66%)	2.988	0.003
Drainage catheter	203 (65.91%)	42 (71.19%)	0.788	0.431
Endotoxic shock	27 (8.77%)	16 (27.12%)	4.015	<0.001
Multiple hospitalizations within 2 years (>2 times)	180 (58.44%)	44 (74.58%)	2.328	0.020
Persistent fungal infection	107 (34.74%)	39 (66.10%)	4.509	<0.001
Serum albumin level^a^ (g/l)	27.50 (23.50, 31.05)	28.00 (24.65, 31.15)	−0.940	0.347
Serum creatinine level^a^ (μmol/L)	59.00 (46.00, 78.00)	63.00 (36.00, 91.00)	−0.035	0.972
Leukocyte count^a^ (10^9^/l)	6.56 (4.40, 10.05)	8.22 (5.18, 10.55)	−1.624	0.105
Total bilirubin level^a^ (μmol/l)	13.30 (7.48, 22.13)	13.80 (9.55, 26.85)	−0.558	0.577
Neutrophil count^a^ (10^9^/l)	5.17 (3.47, 8.09)	6.43 (3.93, 8.41)	−1.153	0.249
Lymphocyte count^a^ (10^9^/l)	0.64 (0.44, 0.95)	0.95 (0.73, 1.26)	−4.245	<0.001
CRP^a^ (mg/ml)	87.40 (49.70, 135.00)	90.45 (34.38, 118.25)	0.421	0.674
PCT^a^ (ng/ml)	0.51 (0.26, 1.86)	0.56 (0.27, 0.99)	0.701	0.483

**Note:**

a Described by median and quartile, and the statistic was the Z value; other items were described as numbers (n - %) and the statistic was the χ2 value.

b Fisher χ2 value.

Patients with multiple fungal infections were older (65 *vs* 61 years, respectively, based on the median age) and had longer hospital stays (55 *vs* 30 days, respectively, based on the median) or ICU stays (19 *vs* 0 days, respectively, based on the median) than patients with a single fungal infection. Furthermore, patients with multiple fungal infections had a higher requirement for invasive mechanical ventilation (64.41%) and central venous catheter (79.66%) compared with patients with a single fungal infection (34.09% and 59.09%, respectively). In addition, patients with multiple fungal infections had a higher predisposition to diabetes (30.51%), endotoxic shock (27.12%) and persistent fungal infection (66.10%) compared with patients with a single fungal infection (10.39%, 8.77% and 34.74%, respectively). Patients with multiple fungal infections had multiple hospitalisations within the past 2 years (>2 times) (74.58%), whereas the number of patients with a single fungal infection with multiple hospitalisations was smaller (58.44%). In addition, 53.57% of patients with a single fungal infection and 27.12% of patients with multiple fungal infections had solid tumours, whereas 59.42% of patients with a single fungal infection and 25.42% of patients with multiple fungal infections had recent surgery (within the past 2 weeks). Furthermore, the lymphocyte count was significantly lower in patients with a single fungal infection (0.64 × 10^9^/L, based on the median) than in patients with multiple fungal infections (0.95 × 10^9^/L, based on the median), with statistically significant differences. Detailed information is provided in Table 3.

### Prediction of risk factors related to death using machine learning

**Figure 4:**
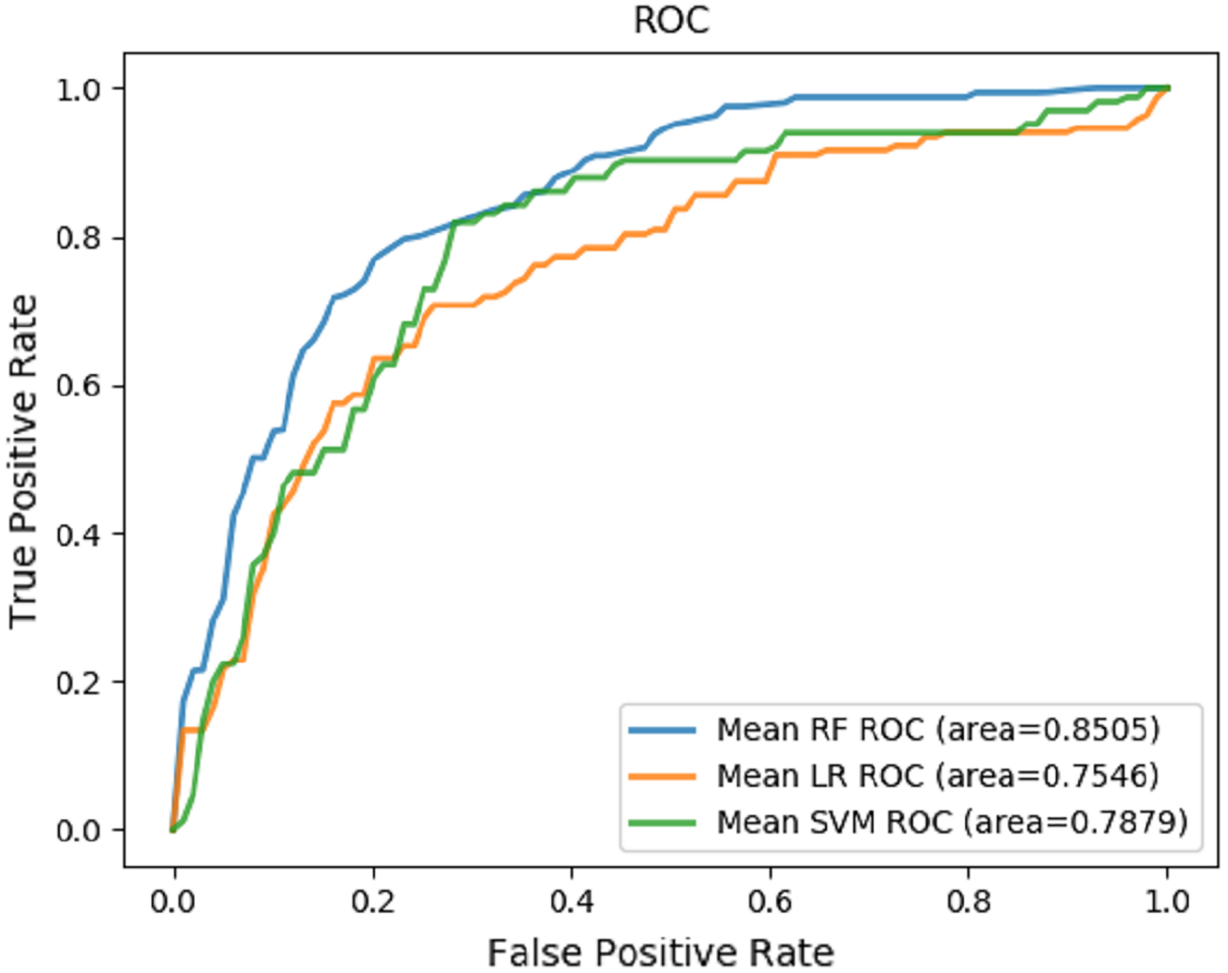
Prediction mode. The ROCs of the prediction of risk factors for death using machine learning mode.

Random forest, logistic regression and support vector machine were used to predict death and evaluate performance (Table 4). The receiver operating characteristic (ROC) curves were generated based on our datasets (Fig. 4). The random forest played an important role in classification and regression and showed excellent performance. It was used to interpret different characteristics of patients and predict the risk factors for candidemia and bacteraemia. The results revealed that serum creatinine level, endotoxic shock, length of stay in ICU, age, leukocyte count, total parenteral nutrition, total bilirubin level, length of stay in the hospital, PCT level and lymphocyte count were the most important prognostic factors for concomitant candidemia and bacteraemia. Detailed information is provided in Table 5. The RF model showed satisfactory performance with these 10 characteristics in our datasets, with an AUC value of 0.8505 (Fig. 4).

**Table 4:** Performance of the machine-learning algorithms.

Model	F1 score	Accuracy	Precision	Recall	AUC
Random forest (RF)	0.5787 (+/− 0.1283)	0.7836 (+/− 0.0363)	0.6824 (+/− 0.1007)	0.5904 (+/− 0.1452)	0.8505 (+/− 0.1062)
Logistic regression (LR)	0.5730 (+/− 0.1100)	0.7589 (+/− 0.0910)	0.6419 (+/− 0.1221)	0.5848 (+/− 0.1189)	0.7546 (+/− 0.0559)
Support vector machine (SVM)	0.4630 (+/− 0.0587)	0.7726 (+/− 0.0372)	0.4549 (+/− 0.1531)	0.5065 (+/− 0.0162)	0.7879 (+/− 0.0822)

**Table 5:** Feature importance rank.

Risk factor variables	Importance rank
Serum creatinine level (g/L)	0.066185
Endotoxic shock	0.063646
Length of stay in ICU (days)	0.057055
Age	0.054846
Leukocyte count (10^9^/L)	0.050269
Total parenteral nutrition	0.045256
Total bilirubin level (umol/L)	0.042580
Length of stay in the hospital (days)	0.037216
PCT level (ng/mL)	0.037109
Lymphocyte count (10^9^/L)	0.036857

## Discussion

To the best of our knowledge, researches on concomitant candidemia and bacteraemia are limited. We collected the detailed clinical information of 367 patients with candidemia and bacteraemia from January 2013 to January 2018 in a provincial medical centre in the northeast of China.

Among all the selected patients in this study, 169 (46.0%) patients stayed in the ICU during hospitalisation, 358 (97.5%) patients stayed in the hospital for >10 days and 224 (61.0%) patients had multiple hospitalisations within the past 2 years. Most patients had diseases including hypoproteinaemia (249/367, 67.8%) and solid tumours (182/367, 49.6%). These conditions may be associated with weak immunity and long-term hospitalisation; moreover, *Candida* colonisation may have worsened the condition of these patients (Nami et al., 2019). Some recent studies have reported features similar to those of the abovementioned conditions (Keighley et al., 2019; Koehler et al., 2019). In addition, urinary catheter insertion (285/367, 77.7%), gastric tube insertion (213/367, 58.0%), central venous catheter insertion (229/367, 62.4%), drainage catheter insertion (245/367, 66.8%) and total parenteral nutrition (298/367, 81.2%) were identified as high-risk factors for candidemia and bacteraemia. As shown in previous studies, invasive medical support may be associated with the deteriorating state of patients with candidemia and bacteraemia (Ang et al., 1993; Fisher et al., 2011; Janum & Afshari, 2016; Ala-Houhala & Anttila, 2020).

Furthermore, *Candida parapsilosis* was identified as the most common fungi affecting patients (151/367, 41.1%), followed by *Candida guilliermondi* (123/367, 33.5%), *Candida albicans* (35/367, 9.5%), *Candida tropicalis* (25/367, 6.8%), *Candida glabrata* (20/367, 5.4%), *Cryptococcus neoformans* (9/367, 2.5%), *Candida lusitaniae* (2/367, 0.5%), *Candida krusei* (1/367, 0.3%) and *Streptomyces* (1/367, 0.3%). In addition, 38 bacterial species were isolated, with 16 (16/38, 42.1%) Gram-positive species and 22 (22/38, 57.9%) Gram-negative species. The most common bacterial species was *Acinetobacter baumannii*, affecting 81 (22.1%) patients. *Enterococcus faecium* and *Pseudomonas aeruginosa* were also common species, affecting 49 (13.4%) and 46 (12.5%) patients, respectively, followed by *Escherichia coli* (37/367, 10.1%), *Klebsiella pneumoniae* (31/367, 8.4%), *Staphylococcus aureus* (29/367, 7.9%) and *Staphylococcus epidermidis* (28/367, 7.6%). This study showed that the *Candida* infection pattern was different from that found in other regions, which can be further investigated (Arendrup et al., 2013; Asmundsdottir, Erlendsdottir & Gottfredsson, 2013; Ulu Kilic et al., 2017; Giacobbe et al., 2020).

Based on the results of machine learning in this study, we found that the most important predictors of death in patients with concomitant candidemia and bacteraemia included serum creatinine level, endotoxic shock, length of stay in ICU, age, leukocyte count, total parenteral nutrition, total bilirubin level, length of stay in the hospital, PCT level and lymphocyte count. Studies have reported that serum creatinine levels were increased in *Candida* infection, which may lead to renal dysfunction and increase infection-related mortality (Wong et al., 1982; Bellomo et al., 2017; Ronco, Bellomo & Kellum, 2019; Arase et al., 2020). Endotoxic shock was associated with *Candida* infection and was one of the main causes of morbidity and mortality worldwide; however, different features might be exhibited according to different species and the immune status of patients (Duggan et al., 2015; Eggimann et al., 2015; Poissy et al., 2020). Prolonged ICU stays could lead to a higher risk of complications and death based on the duration of intensive care (Ruping, Vehreschild & Cornely, 2008; Vincent et al., 2009). Age was also an important factor; elderly patients had low immunity, chronic diseases and multi-organ failure, making them susceptible to invasive *Candida* infections such as candidemia (Pluim et al., 2012; Mundula et al., 2019). The leukocyte count was associated with mortality in patients with *Candida* infections including candidemia (Riley & Rupert, 2015). In addition, total parenteral nutrition could increase the risk of complications and death (Ruiz-Ruigómez et al., 2018; Logan, Martin-Loeches & Bicanic, 2020; Poissy et al., 2020). The total bilirubin level was associated with mortality and factors such as age and primary diseases (Yang et al., 2017; Novák et al., 2020). The length of stay in the hospital could also serve as a prognostic indicator and could be influenced by conditions such as individual differences, primary diseases and different treatment strategies. Moreover, the longer the patients stayed in the hospital, the higher the hospitalisation costed (Lü et al., 2018; Zhang et al., 2020b). PCT is a biomarker for infections and can also serve as a promising prognostic indicator in patients with *Candida* and bacterial infections. Some studies have attempted to differentiate between candidemia and bacteraemia based on PCT levels; however, the differentiation remains challenging (Cortegiani et al., 2019; Honore et al., 2020). Besides, the lymphocyte count was a prognostic indicator of infection and was associated with mortality (Hatinguais, Willment & Brown, 2020; Zhang et al., 2020a).

However, this study has some limitations. First, the data was collected from a single-centre medical database; therefore, the results may be affected by geographical differences, specific management of hospitals and regional policies. Second, limited samples and regional differences may influence the outcomes of machine learning. Therefore, multi-centre studies should be conducted to further explore the epidemiological features and prospective risk factors.

## Conclusions

In this study, the most common *Candida* and bacterial species found in patients with concomitant candidemia and bacteraemia in the First Affiliated Hospital of China Medical University were *Candida parapsilosis* and *Acinetobacter baumannii*, respectively. Serum creatinine level, endotoxic shock, length of stay in ICU, age, leukocyte count, total parenteral nutrition, total bilirubin level, length of stay in the hospital, PCT level and lymphocyte count were identified as the main prognostic factors of death. So far as is known, the types of *Candida* infection and bacterial infection are highly regional. There are few studies on candidemia and bacteremia in China, and the studies in this field in Northeast China remain lacking. Our research fills the gap in this part.

## Supplemental Information

10.7717/peerj.13594/supp-1 Supplemental Information 1 Detailed information of 367 patients with candidemia and bacteraemia.

10.7717/peerj.13594/supp-2 Supplemental Information 2 Detailed information of bacteria species.

10.7717/peerj.13594/supp-3 Supplemental Information 3 Detailed information of antifungal susceptibility test.

10.7717/peerj.13594/supp-4 Supplemental Information 4 Computer Code.

10.7717/peerj.13594/supp-5 Supplemental Information 5 Raw data.

10.7717/peerj.13594/supp-6 Supplemental Information 6 Codebook.

## Additional Information and Declarations

### Data Availability

The following information was supplied regarding data availability: The raw measurements are available in the Supplemental Files.
